# A retrospective analysis of the association between tobacco smoking and deaths from respiratory and cardiovascular diseases in the Kassena-Nankana districts of Northern Ghana

**DOI:** 10.1186/s12971-015-0037-8

**Published:** 2015-04-26

**Authors:** Philip Ayizem Dalinjong, Paul Welaga, Daniel K Azongo, Samuel Chatio, Dominic Anaseba, Felix Kondayire, James Akazili, Cornelius Debpuur, Abraham Rexford Oduro

**Affiliations:** Navrongo Health Research Center, Ghana Health Service, Post Office Box 114, Navrongo, Ghana Africa

**Keywords:** Tobacco, Cigarette smoking, Respiratory diseases, Cardiovascular diseases, Verbal autopsy, Navrongo, Kassena-Nankana districts, Ghana

## Abstract

**Background:**

Tobacco use is a public health problem, responsible for approximately six million deaths annually worldwide. It is a risk factor for many diseases including cancers, respiratory and cardiovascular diseases. In low-and middle-income countries, respiratory and cardiovascular diseases are important causes of death. Tobacco use is prevalent in Ghana, but no study had examined the relationship between tobacco use and deaths from respiratory and cardiovascular diseases in the Upper East Region of Northern Ghana. Hence the paper assessed the association between tobacco use and deaths from respiratory and cardiovascular diseases in that region.

**Methods:**

The study used verbal autopsy data collected from the Kassena-Nankana East and West districts of the Upper East Region of Northern Ghana. Data from deceased individuals aged 15 to 59 years whose deaths occurred between January 1, 2004 and December 31, 2011 and with a known cause as well as smoking status were analyzed. Two binary outcome variables were generated from the cause of death data; whether an individual died from respiratory diseases or not, and from cardiovascular diseases or not. Multiple logistic regression models were used to assess the relationship between tobacco use and deaths from respiratory and cardiovascular diseases.

**Results:**

Out of 3,492 deaths with a known cause of death and smoking status, a third of them smoked. About 16.6% of smokers and 8.1% of non-smokers died from respiratory diseases. Approximately, 10.7% of smokers died from cardiovascular diseases compared to 10.6% of non-smokers. In multivariate analyses, individuals with a history of smoking had two-fold increased odds [OR=2.18, 95% CI (1.6-2.9)] of dying from respiratory diseases. Besides, the number of years of smoking as well as the frequency of smoking is significantly associated with deaths from respiratory diseases. No association existed between tobacco use and deaths from cardiovascular diseases.

**Conclusions:**

Within our study we identified a strong relationship between tobacco use and deaths from respiratory diseases, but not an association with deaths from cardiovascular diseases. Our findings highlight the need to make appropriate health interventions to control tobacco use and thus help reduce premature deaths from respiratory and other tobacco linked diseases.

## Introduction

Tobacco use is increasingly a public health problem. Globally, an estimated 6 million people died in 2011 due to smoking [1,2]. It is further projected to kill 50% more people by 2015 than the disease HIV/AIDS [3] and these estimates are expected to rise to 8 million by 2030 if the trend is left unchecked [1]. The burden of tobacco use is gradually shifting from high to low- and middle-income countries [4,5], since the multi-national companies involved in the tobacco industry intend making up for the losses in the advanced countries [2,6,7]. Following this, deaths attributed to tobacco use are expected to reduce by 9% between 2002 and 2030 in the developed countries, but estimated to double from 3.4 million to 6.8 million annually in low- and middle-income countries [3].

Tobacco use is one of the major risk factors for non-communicable diseases [3,8-11] some of which include cardiovascular and respiratory diseases, cancers, etc. Globally, cancer, cardiovascular and chronic respiratory diseases are gauged to be the leading causes of deaths by 2015 [3].

In the context of low-and middle-income countries, cardiovascular diseases are important causes of deaths [12]. According to the WHO, over 80% of the global deaths from cardiovascular diseases occur in low- and middle-income countries, disproportionally affecting the poorest in these settings [13]. A verbal autopsy approach in Vietnam demonstrated that cardiovascular diseases were the leading cause of death in 1999 [14]. Respiratory diseases are also noted to have caused a devastating effect in Africa [15]. Indeed, tobacco users in Africa are expected to die 20 to 25 years earlier from various cancers, respiratory diseases, as well as cardiovascular diseases [16].

Tobacco use is prevalent in Ghana. The common forms of usage include not only cigarette smoking, but also pipe smoking, chewing, sniffing and oral or nasal use [17]. Until recently, there were no studies showing tobacco prevalence for the whole country Ghana [18], most prevalence studies were conducted in the southern urban areas. However, a recent study using data from the 2008 Ghana Demographic and Health Survey found the prevalence of cigarette smoking and other tobacco products to be 7.9% for men and 0.4% for women [18]. As it is in other countries, the evidence demonstrated that tobacco use in Ghana is high among men, rural residents, the poor and those with no formal education [7,18,19].

Geographically, the three regions of Northern Ghana which are considered the poorest, lead in the prevalence rates for cigarette and other tobacco products usage; 31.2% for Upper East Region, 22.5% for Northern Region, and 7.9% for Upper West Region [19]. Despite this high prevalence, no study has documented the relationship between tobacco use and deaths from respiratory and cardiovascular diseases in Northern Ghana, especially the Upper East Region which had the highest prevalence rate. This paper used verbal autopsy data to explore the relationship between tobacco use and deaths from respiratory and cardiovascular diseases among adults aged 15 to 59 years in the Kassena-Nankana East and West districts of the Upper East Region of Northern Ghana.

Verbal autopsy data is used against the background that certification of deaths in Africa is uncommon. According to Adjuik et al*.,* the registration of deaths in Africa is less than 10% compared to the situation in Europe which is almost 100% [20]. Besides, access to health care services is limited and most deaths take place outside health facilities, and thus very challenging to ascertain the cause of death for each individual. But certification of deaths assist to understand the key causes of death in order to take appropriate policy action or implement interventions directed to specific causes. Verbal autopsy is an innovative way of understanding the causes of deaths in the absence of official certification. It is an interview tool that is used in various places and settings to collect data on causes of death [21], especially in settings with poor vital statistics and weak health systems. This paper contributes to our understanding of the relationship between smoking and dying from respiratory and cardiovascular diseases in the study area and other similar settings. This is essential for the development of effective and efficient health policies on tobacco use and the evaluation of existing programs and policies. It will also provide justification for spending on policies on tobacco use by the Government of Ghana.

## Methods

### Study design and setting

The study was cross sectional and carried out in the Kassena-Nankana East and West districts (previously the Kassena-Nankana districts) of the Upper East Region of Northern Ghana. There are approximately 153, 293 residents in the two districts [22]. The districts have a total land area of 1,674 square kilometers and mainly covered by the Sahel and Sudan-Savannah types of vegetation. Topographically, the land area is low-lying with an average height of 1,000 meters above sea level. Average rainfall per annum is 950 mm. In all, there are 216 communities in the districts, of which majority are rural, with only 13% of the population living in urban areas. The main economic activity for the people is agriculture (70%). The districtsare considered among the poorest in Ghana, due to their reliance on subsistence agriculture.

Existing health facilities include 6 health centres, a district hospital, 27 community-based health and planning services (CHPS) compounds, 2 faith-based health facilities and 3 private clinics. In 2007, the doctor-patient ratio was 1:31927 [23]. Malaria, respiratory infections, skin and diarrheal diseases were the four leading causes of death for the year 2008 [23].

### Data

The study used verbal autopsy data to determine the association between tobacco use and deaths from respiratory and cardiovascular diseases. The verbal autopsy data was collected by the Navrongo Health Research Centre through the Health and Demographic Surveillance System (HDSS). The HDSS is a community registration system that collects and updates longitudinal data on births, deaths, pregnancies, marriages and migrations every four months. The HDSS also conducts verbal autopsy for all deaths registered in the surveillance system [22,24]. In 2003, the verbal autopsy tool was modified to collect additional information on the lifestyle of all deceased individuals including their drinking and smoking patterns. The verbal autopsy instrument has both open-and close-ended questions that document information on the signs, symptoms, lifestyle (smoking and drinking patterns) and circumstances leading to death [25,26]. The tool includes a section for verbatim narrations of the circumstances leading to death. The data is usually collected through interviews with close relatives of the deceased using the verbal autopsy instrument. To minimize recall bias by respondents, the interviews are conducted on average 3 months after a death has occurred. Experienced field supervisors are used to carry out the interviews.

Upon completion of the verbal autopsy questionnaire forms, the next stage involved the assignment of possible cause of death by physicians. Three physicians independently review each verbal autopsy questionnaire form and assign an underlying cause of death corresponding to the 3-digit code of the international statistical classification of diseases and health-related problems [27]. If at least two agree on the underlying cause of death, a diagnosis was established. Where there was disagreement among all three, the form was submitted to two additional physicians for review. Where there was verbal autopsy information but no underlying cause of death could be agreed on, the case was declared undetermined. Where little or no information was available to enable an assignment of cause of death, the diagnosis was declared unknown/undetermined.

### Measurement of smoking among the deceased

In measuring the smoking status of the deceased, questions are asked a close relative who provided care for the deceased prior to death, whether the deceased ever used or smoked tobacco during his/her life time. If the response is “yes”, then further questions are asked on the duration of smoking/use of tobacco, frequency, quantity of cigarette smoked/tobacco used, and type of cigarette/tobacco consumed.

### Variables

The main exposure variable for the analysis was the smoking status of the deceased prior to death. On the other hand, the outcome variables for the study were respiratory and cardiovascular diseases. First we determined whether an individual died from respiratory disease or not. If an individual died from respiratory disease, it was categorized as 1, and 0 if otherwise. For this analysis, we classified the following diseases under the category of respiratory diseases; chronic obstructive lung disease, tuberculosis, asthma, acute respiratory infections (including pneumonia and influenza), and other respiratory system illness (including empyema, fistula etc.). The second outcome variable was whether an individual died from cardiovascular disease or not, (1, if the death was associated with cardiovascular disease, and 0 if otherwise). The following diseases were also classified as cardiovascular diseases; hypertensive heart disease, ischemic heart disease, cerebrovascular disease and all other cardiovascular diseases. Other diseases not related to either respiratory or cardiovascular diseases were categorized as “other”.

### Data analysis

**Figure 1:**
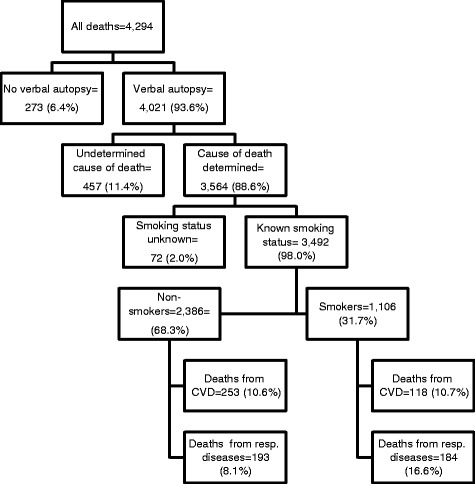
Distribution of deaths for the period 2004–2011.

The analysis was done using STATA 11.2 [28]. All deaths aged 15–59 years and occurring between January 1, 2004 and December 31, 2011 were analyzed. Individuals were more likely to initiate smoking at age 15 and thus the reason for using the age group 15 years and above. Besides, the age range 15–59 years was considered the productive age group. Therefore deaths occurring between that age group imply a greater loss of life expectancy, having a substantial impact on society. Overall 4,294 adult deaths occurred in the specified period. Figure 1 shows the distribution of deaths for the period.

Separate multiple logistic regression models were utilized to assess the relationship between tobacco use and dying from respiratory diseases, and again between tobacco use and dying from cardiovascular diseases. Adjusted odds ratios (OR) with 95% confidence intervals (CIs) were calculated. Reference categories were defined as those usually associated with the lowest risk of dying from respiratory or cardiovascular diseases. Potential confounding variables controlled for were age, sex, place of residence and socio-economic status using wealth index generated from household assets using principal component analysis (PCA). The potential confounding variables were arrived at based on their association with tobacco use and dying from respiratory or cardiovascular diseases from previous studies. Besides, socio-economic status was classified as poorest, poorer, poor, less poor, and least poor. Assets used for the socio-economic computation included both large (for example, land and car ownership) and small household items comprising of radio sets, electric fans, etc.

### Ethics approval

Verbal consent was obtained from household heads or any other senior member of the household before carrying out each update for the health and demographic surveillance system. The update for household members was done every four months which includes the collection of the verbal autopsy data. This consent process had been approved by the Institutional Review Board (IRB) of the Navrongo Health Research Centre.

## Results

### Background characteristics of deaths from respiratory and cardiovascular diseases

Table 1 describes the percentage distribution of the background characteristics of the deaths. Among the deaths with a known smoking status (3,492), approximately 31.7% (1,106) of them were reported to be tobacco smokers preceding their death. About 16.6% of tobacco smokers and 8.1% of non-smokers died from respiratory diseases. Among the smokers, 10.7% died from cardiovascular diseases compared to 10.6% of non-smokers. About 50.8% of men and 2.2% of women were tobacco smokers. Around 12.3% of men and 8.5% of women died from respiratory diseases. There was no marked difference in cardiovascular deaths by sex with 10.4% of men and 11.1% of women dying from cardiovascular diseases. The median age of the deceased was 45 years (IQR: 35–53).

**Table 1:** **Percentage distribution of background characteristics of deaths from respiratory and cardiovascular diseases**

**Variable**	**Number**	**Respiratory diseases**	**Cardiovascular diseases**
	**(%)**		
	**N=3,492**	**n (%)**	**n (%)**
**Smoking status**			
Smokers	1,106 (31.7)	184 (16.6)	118 (10.7)
Non-smokers	2,386 (68.3)	193 (8.1)	253 (10.6)
**Age group**			
15-29	560 (16.0)	33 (5.9)	19 (3.4)
30-49	1,604 (45.9)	160 (10.0)	137 (8.5)
50-59	1,328 (38.0)	184 (13.9)	215 (16.2)
**Sex**			
Female	1,367 (39.2)	116 (8.5)	151 (11.1)
Male	2,125 (60.9)	261 (12.3)	220 (10.4)
**Education**			
No education	1,584 (45.4)	175 (11.1)	194 (12.3)
Primary/JSS	1,336 (38.3)	150 (11.2)	108 (8.1)
Secondary/tertiary	391 (11.2)	30 (7.7)	51 (13.0)
Missing	180(5.2)	22 (12.2)	18 (10.0)
**Place of residence**			
Rural	2,970 (85.1)	340 (11.5)	302 (10.2)
Urban	522 (15.90)	37 (7.1)	69 (13.2)
**Socio-economic status**			
Poorest	751 (21.6)	85 (11.3)	77 (10.2)
Poorer	729 (21.0)	87 (11.9)	71 (9.7)
Poor	749 (21.6)	100 (13.4)	69 (9.2)
Less poor	763 (22.0)	70 (9.2)	83 (10.9)
Least poor	483 (13.9)	4 (7.0)	69 (14.3)
**Frequency of smoking**			
Chain or hourly smoking	177 (5.1)	37 (20.9)	17 (9.6)
Daily smoking	858 (24.6)	138 (16.1)	90 (10.5)
Occasional smoking	71 (2.0)	9 (12.7)	11 (15.5)
No smoking	2,386 (68.3)	193 (8.1)	253 (10.6)
**Duration of smoking**			
1-5 years	531 (15.2)	81 (15.3)	62 (11.7)
6-15 years	73 (2.1)	11 (15.1)	8 (11.0)
>15 years	502 (14.4)	92 (18.3)	48 (9.6)
Non-smokers	2,386 (68.3)	193 (8.1)	253 (10.6)
Total	3,492	377 (10.8)	371 (10.6)

### Association between tobacco use and deaths from respiratory diseases

Table 2 shows the relationship between smoking and dying from respiratory diseases. Individuals who smoked were associated with more than two-fold increased odds of dying from respiratory diseases, in bivariate [OR = 2.31, 95% CI (1.9-2.9)] and multivariate analysis [OR = 2.18, 95% CI (1.6-2.9)]. Likewise, the bivariate analysis indicated that, being older than 30 years, male sex, living in a rural area and a lower socio-economic status was associated with increased odds of dying from respiratory diseases. However, after adjustment for other confounding variables, it was only age that remained significant. Individuals in the age group 50–59 had nearly two-fold increased odds [OR = 1.99, 95% CI (1.3-3.0)] of dying from respiratory diseases. There was no significant association between educational achievement and dying from respiratory diseases.

**Table 2:** **Association between smoking and dying from respiratory diseases among deceased adults (n=3,492)**

**Variable**	**Unadjusted**	**P-value** ^**#**^	*Adjusted	**P-value** ^**#**^
	**OR (95% CI)**		**OR (95% CI)**	
**Smoking status**				
Smokers	2.31 (1.9-2.9)	<0.001	2.18 (1.6-2.9)	<0.001
Non-smokers	1		1	
**Age group**				
15-29	1		1	
30-49	1.8 (1.2-2.7)	<0.001	1.35 (0.8-2.0)	0.002
50-59	2.63 (1.8-3.9)		1.99 (1.3-3.0)	
**Sex**				
Female	1		1	
Male	1.51 (1.2-1.9)	0.006	0.98 (0.7-1.3)	0.904
**Education**				
No education	1.49 (0.9-2.2)			
Primary/JSS	1.52 (1.0-2.3)	0.117	NS	NS
Secondary/tertiary	1			
**Place of residence**				
Rural	1.64 (1.1-2.3)	0.007	1.44 (0.9-2.2)	0.081
Urban	1		1	
**Socio-economic status**				
Poorest	1.61 (1.1-2.5)		1.31 (0.8-2.1)	
Poorer	1.62 (1.1-2.5)	0.009	1.26 (0.8-2.0)	0.216
Poor	2.0 (1.3-3.0)		1.54 (0.9-2.4)	
Less poor	1.29 (0.8-2.0)		1.08 (0.7-1.7)	
Least poor (rich)	1		1	

^#^Overall p-value.

NS – Not significant in bivariate analysis.

^*^Adjusted for age, sex, place of residence and wealth index in separate models.

Missing categories were not included in the regression models.

### Association between tobacco use and deaths from cardiovascular diseases

Table 3 demonstrates the association between tobacco use and deaths from cardiovascular diseases. From the Table 3, there was no statistical significance for tobacco use and deaths from cardiovascular diseases. But in multivariate analysis, being older than 30 years and living in an urban setting was associated with increased odds of dying from cardiovascular diseases. For instance, the age group 50–59 years had over five-fold increased odds [OR = 5.51, 95% CI (3.1-9.2)] of dying from cardiovascular diseases compared to the age group 15–29. In addition, urban dwellers had more than one-fold increased odds [OR = 1.44, 95% CI (1.1-1.9)] of dying from cardiovascular diseases compared to rural dwellers.

**Table 3:** **Association between smoking and dying from cardiovascular diseases among deceased adults (n = 3,492)**

**Variable**	**Unadjusted**	**P-value** ^**#**^	*Adjusted	**P-value** ^**#**^
	**OR (95% CI)**		**OR (95% CI)**	
**Smoking status**				
Smokers	1		1	
Non-smokers	1.02 (0.8-1.3)	0.817	1.16 (0.9-1.5)	0.246
**Age group**				
15-29	1		1	
30-49	2.58 (1.6-4.3)	<0.001	2.59 (1.7-4.9)	<0.001
50-59	5.45 (3.3-8.9)		5.51 (3.1-9.2)	
**Sex**				
Female	1.11 (0.9-1.4)	0.357	NS	NS
Male	1			
**Education**				
No education	1.58 (1.2-2.0)		1.21 (0.9-1.6)	
Primary/JSS	1	0.004	1	0.080
Secondary/tertiary	1.70 (1.2-2.4)		1.49 (1.0-2.2)	
**Place of residence**				
Rural	1		1	
Urban	1.32 (0.9-1.8)	0.058	1.44 (1.1-1.9)	0.020
**Socio-economic status**				
Poorest	1.09 (0.8-1.6)			
Poorer	1.08 (0.8-1.5)			
Poor	1	0.151	NS	NS
Less poor	1.22 (0.9-1.7)			
Least poor (rich)	1.56 (1.1-2.3)			

^#^Overall p-value.

NS – Not significant in bivariate analysis.

^*^Adjusted for age, sex, place of residence and wealth index in separate models.

### Relationship between length, frequency of smoking and dying from respiratory diseases

Individuals who smoked were further classified into groups based on the frequency of smoking and the duration of smoking. Table 4 describes the relationship between frequency of smoking and dying from respiratory diseases, and between duration of smoking and dying from respiratory diseases. This has been examined in two separate regression models. The odds of dying from respiratory diseases increases based on the frequency of smoking. For example, the multivariate analysis showed that chain/hourly smokers had nearly three-fold increased odds [OR = 2.97, 95% CI (1.9-4.6)] of dying from respiratory diseases compared to non-smokers. Those who smoked daily had double the odds of dying from respiratory diseases compared to non-smokers. Occasional smokers were also more likely to die from respiratory diseases compared to non-smokers, but this was not statistically significant.

**Table 4:** **Relative odds of dying from respiratory diseases by frequency and duration of smoking**

**Variable**	**Unadjusted**	**P-value** ^**#**^	**Adjusted** ^*^	**P-value** ^**#**^
	**OR (95% CI)**		**OR (95% CI)**	
**Frequency of smoking**				
Chain/hourly smoking	3.19 (2.2-4.7)		2.97 (1.9-4.6)	
Daily smoking	2.18 (1.7-2.8)	<0.001	2.06 (1.5-2.8)	<0.001
Occasional smoking	1.79 (0.9-3.7)		1.67 (0.8-3.5)	
Non-smokers	1		1	
**Duration of smoking**				
1-5 years	2.13 (1.6-2.8)		2.13 (1.5-2.9)	
6-15 years	2.12 (1.1-4.1)	<0.001	1.87 (0.9-3.7)	<0.001
>15 years	2.54 (1.9-3.4)		2.32 (1.6-3.2)	
Non-smokers	1		1	

^#^Overall p-value; ^*^Adjusted for age, sex, place of residence and wealth index in separate models.

In addition, the number of years of smoking was significantly associated with dying from respiratory diseases. The odds of dying from respiratory diseases increases with increasing number of years an individual smoked. In the multivariate analysis, for instance, individuals who smoked for over 15 years had over two-fold increased odds [OR = 2.32, 95% CI (1.6-3.2)] of dying from respiratory diseases compared to non-smokers.

## Discussion

This study has illustrated that among the deaths recorded for the period in the study area, 31.7% smoked. This is consistent with the results of a recent study conducted in Ghana. Data from the World Health Organization’s (WHO) multi-country study on global aging and adult health (SAGE) was analyzed and it revealed that tobacco prevalence in the Upper East Region (the region for our study), was 31.2% [19]. The finding might be explained by the fact that rural areas usually have high prevalence rates for tobacco use compared to semi-urban and urban places [29,30]. For example, Chockalingam et al. reported that tobacco use was significantly higher for rural residence (23.7%) compared to semi-urban (20.9%) and urban locations (19.4%) [31].

The study also demonstrated that smokers who died from respiratory and cardiovascular diseases were 16.6% and 10.7% respectively. This correlates with other studies as well. Tobacco linked respiratory diseases were shown to be responsible for about 22.1% of deaths in Canada for the year 1998 [32]. In the case of cardiovascular diseases, Pérez et al. reported that deaths from cardiovascular diseases among smokers in 1995 and 2007 respectively, were 52% and 42% in Cuba [33]. However, the proportions reported in their study are far higher than that reported in this paper. According to the authors, their finding put Cuba in the lead globally for deaths attributable to tobacco use.

Besides deaths from respiratory diseases were found to be significantly related to smoking as reported in other studies [15,34,35]. In the multivariate analysis, smoking was still found to be a significant risk factor for deaths due to respiratory diseases. This is supported by other studies as well. In Bangladesh, for instance, tobacco use was associated with 1.58 increased odds (99% CI 0.9-2.8) of dying from respiratory diseases [36]. Our findings showed that chained or frequent smokers (people who smoked every hour) had higher chances of dying from respiratory diseases, compared to non-smokers. The results clearly showed a significant dose–response relationship between dying from respiratory diseases and the quantity/amount of tobacco smoked [37,38]. A similar dose response relationship was observed between the number of years an individual smoked and deaths from respiratory diseases. Thus, the study has demonstrated that tobacco use is a major risk factor for deaths due to respiratory diseases. Therefore tobacco control should be considered a top priority in Ghana.

However, the study could not establish any significant relationship between tobacco use and deaths from cardiovascular diseases. Other studies exist to support our finding. For instance, a prospective study conducted in Bangladesh could not establish any clear association between smoking and deaths from overall cardiovascular diseases [38]. Again, the Singapore Chinese Health Study among men and women of Chinese ethnicity found no apparent relationship between smoking and deaths from stroke [39]. Contrary to our finding, other studies have found a significant association between smoking and deaths from cardiovascular diseases [11,40,41]. Further research in that direction is recommended, especially from other INDEPTH Network sites which collects verbal autopsy data.

Age especially is one of the most important factors determining deaths from cardiovascular diseases. From this study, older individuals as well as individuals living in urban areas had increased odds of dying from cardiovascular diseases. Similar findings exist elsewhere to support our finding. Jiang et al. found that deaths resulting from coronary heart disease in Tianjin (China) were higher among older age groups and individuals living in urban areas [42]. However, it is worrying to realize that people in their prime ages or older are dying due to cardiovascular diseases. The impact of these deaths are enormous on the family, employer and the community due to the fact that these people are either the bread winners for their families, the most experienced employees, or sometimes leaders of their communities [43]. There is the need for education on lifestyle habits (for example, dieting, alcohol consumption, physical inactivity, etc.) especially for older individuals as well as urban dwellers.

### Study limitations

The use of the verbal autopsy tool for data collection has its limitations including accuracy and recall bias by the respondents. It is a challenge determining whether the caregiver responding for the deceased is truthful in his/her responses. Again, it is possible for non-smokers to have died from tobacco related diseases (both respiratory and cardiovascular) due to exposure to second hand smoke. Our study would not have captured such deaths since the main exposure variable for the analysis was the smoking status of the individual prior to his/her death. Therefore our findings on the association between smoking and deaths from both respiratory and cardiovascular diseases could have been underreported.

Nonetheless, the study was a population based study with vigorous study oversight, well trained field staff for data collection, and a well-defined health and demographic surveillance system for monitoring deaths and other key events. Again, the verbal autopsy tool for the study was developed and standardized by the WHO and the International Network for the Demographic Evaluation of Populations and Their Health (INDEPTH Network).

### Policy recommendations

In most sub-Saharan African countries including Ghana, the challenges of tobacco use are emerging, but stringent policies on smoking and use of tobacco products are minimal and not effectively enforced. From the findings of this study, it is clear that smoking is a significant predictor of deaths from respiratory diseases. This therefore, calls for urgent need to re-assess the state of the implementation of the Framework Convention on Tobacco Control (FCTC) to reduce tobacco use among the adult population. In Ghana particularly, the Public Health Bill incorporating the Tobacco Control Bill requires quick action to become a Legislative Instrument (LI) to allow for its enforceability. The provisions of the Tobacco Control Bill if well enforced will contribute to reducing respiratory and other tobacco related diseases, thus minimizing premature deaths in the country. Following our findings from this study, which took place in a predominantly rural area, there is the need to enact policies addressing tobacco use in both rural and urban areas.

## Conclusions

The analysis showed an association between tobacco use and deaths from respiratory diseases in the Kassena-Nankana East and West districts. A significant relationship was observed for tobacco use and deaths resulting from respiratory diseases. Tobacco users were more prone to dying from respiratory diseases compared to non-users. Our study could not establish any significant relationship between tobacco use and deaths from cardiovascular diseases. Only age and urban location were found to be strong predictors of deaths from cardiovascular diseases. There is need to adopt appropriate health interventions to control tobacco use. Controlling tobacco use can contribute to reducing respiratory and other tobacco linked diseases and by extension deaths related to tobacco usage.

## Abbreviations

CI: Confidence Interval
FCTC: Framework Convention on Tobacco Control
HDSS: Health and Demographic Surveillance System
LI: legislative instrument
INDEPTH Network: International Network for the Demographic Evaluation of Populations and Their Health
IRB: Institutional Review Board
OR: Odds Ratio
PCA: Principal Component Analysis
WHO: World Health Organization
